# Survey data assessing the junior high school students’ learning attitudes toward home-based education amidst the Covid-19 pandemic

**DOI:** 10.1016/j.dib.2023.109241

**Published:** 2023-05-16

**Authors:** Dr. Saniya G. Abirin

**Affiliations:** Integrated Laboratory High School Western Mindanao State University Normal Road, Baliwasan, Zamboanga City, Philippines

**Keywords:** Students’ learning attitudes, Academic performance, Students’ motivation, Junior high school demographics, Junior high school population

## Abstract

This dataset was obtained to determine the learning attitudes of junior high school (JHS) students toward Home-Based Education (HBE). A descriptive - survey approach was used employing a proportional stratified random sampling to determine the samples *n* = 398 drawn from a total population of *N* = 75,542 junior high school students enrolled in 42 public secondary schools in Zamboanga City Division for SY 2020–2021. The data collection was conducted from August 2021 to September 2021 amidst lockdown thus, a combined data collection - Online & Offline was conducted using an adopted validated instrument. Out of 398 samples considered, only 383 eligible consenting JHS students completed the survey with a response rate of 96.23% to include 274 (71.54%) Online & 109 (28.46%) Offline. There were two problems investigated to include determining the learning attitudes of JHS students measured in terms of Nature, Anxiety, Expectations, & Openness to Learning, and to determine whether there exists a significant difference between the learning attitudes of junior high school students across four Independent Variables (IVs): gender, grade level, age and socioeconomic status (SES). Mean, Standard Deviation, and MANOVA were used to analyze the data gathered. Data assumptions were employed before employing MANOVA, and based on the results obtained from the data analysis, the overall learning attitudes of junior high school students toward HBE is High; and learning attitude varies significantly across grade level and age in terms of Nature and Anxiety of Learning, and in the SES in terms of Expectations of Learning. .

**Specifications Table**

**Table:** 

Subject	Social Science
Specific subject area	Secondary Education
Type of data	Table
How the data were acquired	Before the start of data collection in 42 schools with a total population of *N* = 75,542, a permission letter was sent to the Office of the Schools Division Superintendent (SDS) of Zamboanga City seeking approval for the conduct of the data collection. An ethics clearance secured from the Ethics Review Committee was also submitted to the Office of the SDS to ensure that the research protocol has duly undergone a thorough review before the data collection. Following the approval, the researcher received an endorsement letter coming from the Office of the SDS granting permission to do research among 398 junior high school students across 42 schools under the said division. With this, the researcher asked the list of secondary school Principals from the planning section office to officially coordinate with the latter about the data collection. In addition, the master list of JHS enrolment data for SY 2020–2021 was also provided by the planning office to the researcher. Following the basic Covid-19 health and safety protocols, the endorsement letter coming from the Office of the Schools Division Superintendent (SDS) of Zamboanga City was presented to each of the School Principals of the 42 schools. During the said visit, important details about the data collection were discussed such as the target respondents, the required number of samples, and the mode of data collection. To select the samples from each school, the curricular chair or adviser of each school provided the list of students to the researcher. The list of students including the demographic profiles was provided. Using the list, a sampling frame for each school was created. In creating the sampling frame, the existing Covid-19 situation in the City was considered alongside the well-representativeness of the samples to the target population. The sampling frame created for the sample selection included all junior high school students who were permitted by their parents to participate in the data collection, and at the same time students who met the other criteria set by the researcher. Since only the eligible consenting students were included in the sampling frame, the researcher asked for the adviser or curricular chairperson's help in distributing the copy of the parent's consent together with the informed consent and student's assent to determine consenting students. The printed copies of such were distributed to all the parents or guardians during the module distribution or outputs submission in school, and a digital copy was sent to all parents or guardians who could be accessed via FB Messenger. The informed consent and student assent were thoroughly discussed in the language that could be best understood by the parent/guardian while emphasizing the voluntary participation of their child. The parents/guardians and students were then asked to sign or put a mark on the parent's consent and student's assent respectively to confirm their willingness to participate in the data collection. All
	accomplished parent's consent and students’ assent were collected by the adviser, and all the names of eligible consenting students were listed and encoded into Microsoft Excel to constitute the sampling frame for each school. A random formula was applied to assign a unique code for each student, and after assigning codes, the list is shuffled and filtered to pool the samples needed. Since the data collection was conducted amidst lockdown restricting students to leave their residence, the data collection was carried out using a mixed-method approach to include online and offline. According to Dillman et al. (2014) and Singleton and Straits (2009) as cited by Ponto [17], using a combination of methods of survey administration can help ensure better sample coverage by providing all individuals an equal chance of inclusion in the sample, therefore, can reduce coverage error. Before the data collection, the adopted survey questionnaire underwent pilot testing among 16 junior high school students coming from two public schools, Of the 16 students who participated in the pilot testing of the questionnaire, three students came from Grade seven, five students from Grade eight, six students from Grade nine, and two students from Grade 10. According to Fink (1995) in Sang, Mail, Abd Karim, Ulum, Mufli, and Lajuni (2017), the minimum number for a pilot test in most student questionnaires is 10. This claim is supported by Hill (1998) in Tappin [19] who suggests that 10 to 30 participants are needed for pilot tests in survey research. A reliability analysis using Cronbach's Alpha was run to obtain the alpha reliability coefficient, and based on the result, the adopted survey questionnaire is valid for use by junior high school students within the context of the current data collection. The computed reliability coefficients obtained for the students’ attitude survey questionnaire was 0.832 which is greater than 0.70, according to Hair, Bush and Ortinau, (2003) in Sang et al. [18] were acceptable, therefore no changes were made in the questionnaires. Before its distribution, the hard copy of the survey questionnaires for offline and the Google form for online was checked first for any defect such as incompleteness, unreadability, broken links, as well as the functionality of the required option buttons in the Google Form. Survey questionnaires including the Google link for online and offline participants were distributed to the Curricular chair or adviser together with the list of eligible consenting students who were randomly chosen to participate in the .data collection. A total of 398 students to include 283 for online, and 115 students for offline received the Google link and printed survey questionnaires respectively. Since students were not allowed to go out, the parents or guardians of the selected consenting students in 25 schools who participated offline were contacted to get the questionnaires at school, and those students under the 17 schools who participated online was contacted via their own FB messenger or cellphone number of parents/guardian to inform them about the survey link being sent to their account. Both surveys for offline and online were answered by the students at home. Upon completion, the survey questionnaires were returned by the parents or guardians to the adviser or curricular chair at school, while for online, submission of responses was done via Google sheet. Out of 398 students who were selected as participants in the data collection, 15 students failed to complete the survey making the response rate at 96.23% with a total of 383 students to include 274 (71.54%) students who participated online and 109 (28.46%) students who participated offline.
Data format	Raw Analyzed Filtered
Description of data collection	The data collected from the 383 high school respondents were inputted into Statistical Social Science Packages (SPSS) for data analysis. Before treating the data, data assumptions were carried out to determine the appropriateness of the inferential statistical tools to be used. Data screening involved checking the assumptions of MANOVA to include normality, homogeneity of variance, homogeneity of covariance, and outliers to explore the appropriateness of the data for MANOVA. In terms of normality, a histogram and the absolute values of skewness and kurtosis were used to assess the normality of the data for actual samples. This is in
	accordance with Kim [12] that sample sizes greater than 300, a histogram, and the absolute values of skewness and kurtosis without considering z-values can be used. Either an absolute skew value larger than 2 or an absolute kurtosis larger than 7 may be used as reference values for determining substantial non-normality (Kim, 2003*;* Kline, 2010 in [20]). In addition, according to Hartmann et al. [9], data can be assumed to be normally distributed since the sample size is greater than 30. This assumption is backed up by the Central Limit Theorem, which states that for a large sample size (*n*>30), the sampling distribution is approximately normal, irrespective of the shape of the population distribution (Mann 2012 in [3,9]. The sample size is usually considered to be large if *n* ≥ 30 ([9]; Pallant, 2007). This claim is supported by Altman and Bland (1995) in Ghasemi and Zahediasl [7] that if the data consists of hundreds of observations, the distribution of the data can be ignored. This implies that parametric procedures can be applied even when the data are not normally distributed (Elliot and Wood Ward, 2007). The second assumption that was carried out was in respect to homogeneity of variance, wherein it suggests that all data should have the same or similar variances [16]. For checking this assumption, Box's M was used since group sizes were over 30. According to Kelly [11] and Allen and Bennet [1], if group sizes are over 30, then the MANOVA is robust against violations of homogeneity of variance-covariance matrices assumption. This implies that a one-way MANOVA can still be applied despite the violated assumption about the homogeneity of variance-covariance among the dependent variables. Moreover, the outlier analysis was also carried out to assess whether data are coherent to multivariate analysis. Outliers are sample units with extreme values that have an unusual combination of values for more than one variable (multivariate outliers). Outliers can be a matter of concern because they can have a large effect on the outcome of an analysis [15]. In this case, the Mahalanobis distance (MD) was the metric used for testing multivariate deviation where each observation obtains a numerical measurement to the centroid of the data set and the correlation of the data sets is taken into account via the covariance matrix [6]. The MD was used to check the data set by creating p-values using the Chi-Square function. Each dependent variable was analyzed and scored separately by creating a column of p-values at the end of the data set. The critical value of chi-square at *p* < 0.001 was used for the calculation of Mahalanobis Distance with degrees of freedom (df). This assumption is backed up by Mertler and Reinhart (2017) and Finch (2012) as cited by Welch and Areepattamannil (2016) that the accepted criterion for outliers is a value for Mahalanobis Distance that is significant beyond *p* < 0.001 [8]. In summary, results for the assumption testing indicated that data were normally distributed with values for skewness and kurtosis less than 2 and 7 respectively (Kim, 2003*;* Kline, 2010 in Welch and Areepattamannil, 2016) and with a sample size which is greater than 30, data assumed normality irrespective of the shape of the population distribution ([3,9]; Altman and Bland, 1995 in [7]); and no outliers were beyond *p*<0.001 [8], thus MANOVA was applied.
Data source location	• Institution: 42 Public Secondary Schools • City/Town/Region: Zamboanga City, Region IX • Country: Philippines
Data accessibility	Repository name: Mendeley Data Data identification number: **CC BY 4.0** Direct URL to data (SAV file): https://data.mendeley.com/datasets/f887wrzymk/1 Direct URL to Learning Attitudes’ Instrument and raw data excel file, and supplementary materials: https://data.mendeley.com/datasets/ftnbg9pptz

## Value of the Data

• This dataset that aims to assess the learning attitudes of junior high school students was obtained using a descriptive-survey employing a proportional stratified random sampling from 383 respondents drawn from a total of 75,542 population junior high school students enrolled across 42 public secondary schools in Zamboanga City, Philippines for School Year 2020 – 2021 amidst the Covid – 19 pandemic, where individuals below 21 years old were restricted to leave their residence.
• This dataset which was obtained using an adopted 42-item validated survey questionnaire from Kara [10] can be used to assess the learning attitudes of junior high school students in terms of their nature of learning, anxiety of learning, openness to learning, and expectations of learning toward Home-Based Education across gender, grade level, age, and socioeconomic status.
• Data can be used as basis for policy formulation on the implementation of Home-Based Education as a viable learning modality in the new normal in Zamboanga City, Philippines.
• Future researches using a different research design across other variables such as religion, higher educational level such as college can be employed to further assess the learning attitudes of students toward Home-Based Education.

## 1 Objective

This dataset measured in terms of the students’ Nature of Learning, Anxiety of Learning, Expectations of Learning, and their Openness to Learning toward Home-Based Education aimed to assess the learning attitudes of high school students in Zamboanga City, Philippines toward Home-Based Education amidst the Covid-19 pandemic.

***Nature of Learning*** refers to students’ perception of learning; ***Anxiety of Learning*** refers to the anxiety that high school students experienced towards HBE such as problems in concentrating, being fed up, forgetfulness, boredom, feeling of not learning, and the learning difficulties experienced during HBE; ***Expectations of Learning*** refers to what and how students expect to learn during HBE, and the impact of HBE to them during learning; and ***Openness to Learning*** refers to high school students’ feeling of excitement, optimism, and the positive attitude towards HBE.

This dataset was collected from the 383 eligible respondents out of 398 samples considered during data collection. Those responses coming from the other 15 JHS students were considered invalid and were not included in the data analysis due to the incomplete provision of the necessary data needed in the data analysis.

In order to select samples, a proportional stratified random sampling was used covering the total population of 75,542 junior high school students enrolled across 42 public secondary schools in Zamboanga City, School Year 2020–2021. In determining the sample size, a Slovin's Formula was used, and based on the computation, a sample size *n* = 398 at 95% confidence interval and 5% margin of error was obtained. To obtain a proportional sample for each stratum to the population size of each school, first, the 42 schools were considered as strata with four demographic variables such as gender, grade level, age, and socioeconomic status. Each of these 42 schools with its respective population size was identified through the SY 2020 – 2021 JHS enrolment data provided by the planning section of DepEd Zamboanga City (ZC) Division.

## 2 Data Description

The dataset which assesses the learning attitudes of junior high school (JHS) students toward Home-Based Learning and the learning attitudes measured across gender, grade level, age, and socioeconomic status is available in SAV format (1).

The learning attitudes was measured in four dimensions namely Nature of Learning, Anxiety of Learning, Expectations of Learning, and their Openness to Learning. These four dimensions of learning were measured using an adopted Students’ Attitude Survey Questionnaire (https://data.mendeley.com/drafts/ftnbg9pptz) with items number one to seven to measure **Nature of Learning**; items number eight to 20 to measure **Anxiety of Learning**; items number 21 to 29 to measure **Expectations of Learning;** and items number 30 to 40 to measure **Openness to Learning using** a five-point Likert Scale 1-Never, 2-Rarely, 3- Sometimes, 4-Often, and 5-Always.

Employing a cross-sectional descriptive-survey design approach, this dataset was obtained from a total of 383 valid responses out of 398 samples from grades seven to 10 considered during the data collection. Responses from the 15 high school students were not included due to incomplete provision of necessary data needed for the data analysis.

For Table 1, students’ responses were analyzed using Mean and Standard Deviation and interpreted using Mean Score interpretation scale 1.00 – 1.79 Very Low; 1.80 – 2.59 Low; 2.60 – 3.39 Moderate; 3.40 – 4.19 High; 4.20 – 5.00 Very High adopted from Kuntiyawichai et al. [14] and Kitjaroonchai [13].

**Table 1:** Students’ learning attitudes towards home-based education (*n* = 383).

	Mean	SD	Learning Attitude
Nature	3.08	0.57	Moderate
Anxiety	3.31	0.64	Moderate
Expectations	3.98	0.71	High
Openness	3.84	0.69	High
Learning Attitudes	3.56	0.49	High

The provided descriptive statistics are shown in Table 1 and the Multivariate Analysis is shown in Table 2. Supplementary material which contains the informed consent, student's assent and full questionnaire that were provided to participants during data collection are available in this link (see https://data.mendeley.com/datasets/ftnbg9pptz).

**Table 2:** Test-of-between subjects: students’ learning attitudes across gender, grade level, age, and SES.

IVs	DVs	Groups	n	Mean	SD	df	F	p
Gender	Nature	Male	187	3.115	0.598	1	1.765	.185
		Female	196	3.039	0.532			
	Anxiety	Male	187	3.278	0.683	1	.868	.352
		Female	196	3.339	0.588			
	Expectations	Male	187	3.908	0.757	1	3.709	.055
		Female	196	4.048	0.661			
	Openness	Male	187	3.784	0.734	1	2.682	.102
		Female	196	3.899	0.641			

Grade Level	Nature	Grade - 7	102	2.924	0.532	3	3.822	.010
		Grade - 8	101	3.082	0.559			
		Grade - 9	99	3.165	0.601			
		Grade - 10	81	3.152	0.541			
	Anxiety	Grade - 7	102	3.135	0.609	3	5.454	.001
		Grade - 8	101	3.270	0.710			
		Grade - 9	99	3.384	0.638			
		Grade - 10	81	3.488	0.506			
	Expectations	Grade - 7	102	3.924	0.728	3	.989	.398
		Grade - 8	101	3.920	0.765			
		Grade - 9	99	4.027	0.659			
		Grade - 10	81	4.066	0.687			
	Openness	Grade - 7	102	3.871	0.709	3	.371	.774
		Grade - 8	101	3.781	0.767			
		Grade - 9	99	3.866	0.667			
		Grade - 10	81	3.857	0.591			

Age	Nature	14 y/o & Below	193	3.018	0.576	1	4.172	.042
		15–24 y/o	190	3.135	0.550			
	Anxiety	14 y/o & Below	193	3.226	0.669	1	6.740	.010
		15–24y/o	190	3.394	0.591			
	Expectations	14 y/o & Below	193	3.969	0.709	1	.075	.784
		15–24 y/o	190	3.989	0.717			
	Openness	14 y/o & Below	193	3.899	0.670	1	2.584	.109
		15–24 y/o	190	3.786	0.706			
Socio-Economic Status	Nature	Below 10,000	285	3.056	0.586	1	1.387	.240
		10,000 & Above	98	3.134	0.501			
	Anxiety	Below 10,000	285	3.287	0.627	1	1.339	.248
		10,000 & Above	98	3.374	0.663			
	Expectations	Below 10,000	285	3.919	0.748	1	8.080	.005
		10,000 & Above	98	4.154	0.563			
	Openness	Below 10,000	285	3.811	0.724	1	2.398	.122
		10,000 & Above	98	3.936	0.571			

Table 2 shows the Multivariate Analysis (MANOVA) to examine whether there exists a significant difference in the Students’ Attitudes measured in terms of the four dimensions namely Nature, Anxiety, Expectations, and Openness to Learning toward Home-Based Education when the variable is grouped according to gender, grade level, age, and socioeconomic status. The independent variables namely **Gender** was measured **using** a self-report measure on gender orientation such as male or female; **Age** was measured using a self-report measure for two age groups Youth (≤14 years old), and Young Adult (15 years old – 24 years old) following the United Nations guideline (1982) in Caceres, Melo, Santos (2013); **Grade Level** was a self-report measure from Grades seven to ten; and **Socio-Economic Status was** a self-report measure of the income of parents/guardians measured based on two-income classifications Poor (monthly income <₱10,481) and Low Income to Rich (monthly income ≥ ₱10,481) following the SES classification by PSA (2020) as cited by Albert (2019). These figures were rounded off to ₱10,000 to give a better estimate.

Before conducting MANOVA, data assumptions were carried out in order to test the appropriateness of MANOVA in treating the data.

## 3 Experimental Design, Materials and Methods

An ethics clearance and permission to collect data across 42 secondary schools were sought from the Ethics Review Committee and Office of the Superintendent respectively. Upon approval, the researcher set a schedule to visit each of the 42 secondary schools.

The data were gathered from all secondary students from grades 7 to 10 enrolled in 42 schools for SY 2021–2022 with a total population of 75,542. A proportional stratified random sampling was employed across gender, grade level, age, and socioeconomic status. In selecting samples, Slovin's formula was used, and a 398 sample size was obtained. Only students who met the criteria set by the researcher and consented by their parents were included in the sampling frame. Samples were randomly drawn from the sampling frame consisting of consenting students. This less stringent criterion was employed to ensure the safety of students from the threat of Covid-19. According to Kelly, Clark, Brown, Sitzia (2003) in AlQotba, Al Nuaimi, Al Mujalli et al. [2], the Covid-19 pandemic may “serve as an excuse for using less stringent criteria in choosing samples without assessing the extent of bias introduced during the survey process”.

Data collection was conducted amidst lockdown due to the increasing cases of Covid-19, thus, it was done in two ways: online and offline utilizing an Attitude Survey Scale adopted from Kara [10] (See https://data.mendeley.com/datasets/ftnbg9pptz). According to Dillman, Smyth, and Christian (2014) and Singleton and Straits (2009) as cited by Ponto [17], “using a combination of methods of survey administration can help ensure better sample coverage by providing all individuals an equal chance of inclusion in the sample, therefore, can reduce coverage error”. The instrument was prepared similarly in printed and digital form. Settings were set in Google form to enable one response only from the respondents and to avoid duplicates in responses. To test the appropriateness and reliability of the instrument for the current data collection, the adopted instrument was pilot tested among 16 secondary students. According to Fink (1995), as cited by Sang et al. [18]), “the minimum number required for pilot testing in most student questionnaires is 10″. This claim is supported by Hill (1998) in Tappin [19] who suggested that 10 to 30 participants are needed for pilot tests in survey research. Based on the result obtained, a high degree of consistency between the given items was observed with Cronbach's alpha 0.832 obtained for the Students’ Learning Attitudes Scale. This result is supported by Hair, Bush, and Ortinau (2003) in Sang, Mail, Karim, Ulum, Mufli, and Lajuni (2017) who stated that the widely accepted cutoff for an instrument is Cronbach's alpha higher than 0.70. Thus, no further changes were needed, and the adopted Students’ Learning Attitudes scale was used in the actual data collection.

A total of 283 respondents participated in an online survey and 115 students in the offline survey which summed up to 398. All these 398 respondents received the Google link and printed questionnaire respectively. Out of 398 samples, 15 students failed to complete the survey which made the response rate 96.23%. According to Fincham [5] “this response rate approximating 60% is what any research should achieve, and a response rate of ≥80% must be achieved as the standard for evaluation for the journal”.

The data collected from the 383 high-school respondents were inputted into Statistical Social Science Packages (SPSS) for data analysis. Before treating the data and to protect its integrity, data assumptions were carried out to determine the appropriateness of the inferential statistical tool to be used (Tabachnick and Fidell, 2007 as cited by [4].) Data assumptions testing results indicated that data were normally distributed with skewness and kurtosis values less than 2 and 7 respectively ([12]*;* Kline, 2010 in Welch and Areepattamannil, 2016). Moreover, according to Hartmann, Krois, Waske, [9], Barr et al. [3], Altman and Bland (1995) as cited by Ghasemi and Zahediasl [7]), “for a sample size greater than 30, data are considered to assume normality irrespective of the shape of the population distribution”. Further, since no outliers were beyond *p*<0.001 [8], thus the parametric procedure was applied.

## 4 Data Analysis

Data were collected using an adopted validated survey questionnaire from Kara [10]. In order to ensure its appropriateness for the existing data collection, this questionnaire was pilot tested among 16 high school students with similar characteristics with the target respondents. The result of the pilot testing indicated that the survey questionnaire used to collect data was valid and reliable with Cronbach's alpha 0.832 which is interpreted as acceptable.

This survey questionnaire was distributed via offline and online among 398 students drawn from 75,542 total population. Those students who were reachable via face-to-face survey answered a printed survey questionnaire, while others were surveyed using a Google form.

A total of 398 students to include 283 for online, and 115 students for offline received the Google link and printed survey questionnaires respectively. Out of 398 students who participated in the data collection, only 383 were included in the data analysis due to theincomplete provision of the necessary data coming from the other 15 respondents. These incomplete data were considered invalid and excluded from the data analysis.

Data were analyzed using Mean and Standard Deviation to measure the Learning Attitudes of high school students in terms of Nature of Learning, Anxiety of Learning, Expectations of Learning, and Openness to Learning. Mean scores obtained were interpreted using the scale 1.0–1.79 Very Low, 1.80–2.59 Low, 2.60–3.39 Moderate, 3.40–4.19 High, and 4.20–5.00 Very High.

On the other hand, in order to determine whether there is a significant difference in the Learning Attitudes of high school students measured in terms of four dimensions namely: Nature of Learning, Anxiety of Learning, Expectations of Learning, and Openness to Learning when the variable is categorized according to gender, grade level, age, and socioeconomic status respectively, Multivariate Analysis of Variance (MANOVA) was usedData assumptions were conducted to determine the appropriateness of MANOVA to treat data.

## Ethics Statements

All junior high school students from whom data were collected were provided with student's assent and informed consent containing the purpose of data collection, the target respondents, the voluntary participation, confidentiality, data collection related risks, compensation, and benefits. Informed consent (see https://data.mendeley.com/datasets/ftnbg9pptz) was prepared in English and translated into three dialects to be easily understood by the respondents and the parents. An ethics clearance with Protocol Code: 2021 – 0059 dated July 26, 2021 was sought from the Ethics Review Committee in an accredited school in Zamboanga City making sure that the highest ethical principles were in place during data collection. Below were the ethical considerations considered before, during, and after data collection was conducted.

Before collecting data, the data collection protocol was submitted for an Ethics Clearance to ensure that it met safety requirements for respondents and that the necessary elements were included in the informed consent documents and followed throughout the data collection. All recommendations given by the institutional review board were implemented to ensure that data collection was conducted with the highest ethical principles in place and that no data were collected until approval from the ethics board.

All respondents included in the data collection were only permitted to participate after being granted parental consent. The parental consent that came with informed consent was provided to the parents/guardians of the respondents to inform them about the data collection. It included identification of the researcher; an indication of how the participants are selected; identification of the purpose of the research; identification of the benefits for participating; identification of the level and type of participant involvement; notation of risks to the respondent; guarantee of confidentiality to the respondent; assurance that the respondent can withdraw at any time; and provision of names of persons to contact if questions arise. The informed consent was provided with a translation made by professionals and native speakers of the dialect to include Tausug, Visayan, and Chavacano for the parent/guardian to be made fully aware of what the research data collection was all about and how the data collection should be carried out.

Since data collection was done amidst the rising cases of Covid-19 infections, parental consent and students’ voluntary participation were sought before allowing any of the respondents to participate in the data collection. Both students and parents were fully informed that students’ decision to participate during data collection procedure was entirely voluntary and that they can refuse to take part in or may withdraw their participation at any time without penalty or loss of benefits to which they are entitled to. The potential respondents were also assured that they may discontinue the data collection for any reason.

Moreover, the possible risks that the respondents may experience during data collection were also addressed by employing some strategies or procedures that may minimize these possible risks such as but not limited to designing the survey questionnaire appropriate for the level of the respondents in terms of language use, format, and construct and was answerable only in 20–40 min or less.

In terms of confidentiality, all the information associated with the high school respondents that may disclose their identity was stored in a password-protected storage devices such as USB, and external hard drives or a secured file cabinet for those hard copies of accomplished survey questionnaires. Moreover, additional measures were taken to ensure that the information provided by the respondents remains anonymous by replacing all names of all respondents, with pseudonyms to minimize the potential that a respondent can be identified in the data. Moreover, data were kept only until the duration of the data collection- related activitiesand were physically destroyed by employing disk shredding or burning as soon as the data collection-related activities have been completed.

## Declaration of Competing Interest

The author declares to have no known competing financial interests or personal relationships which have, or could be perceived to have, influenced the work reported in this article.

## Data Availability

STUDENTS' LEARNING ATTITUDES DATASET AND SUPPLEMENTARY MATERIALS (Original data) (Mendeley Data). LEARNING ATTITUDES OF JHS STUDENTS (Original data) (Mendeley Data). Learning Attitudes of Junior High School Students Toward Home-Based Education (Original data) (Mendeley Data). STUDENTS' LEARNING ATTITUDES DATASET (Original data) (Mendeley Data)
